# The antecedents of oral care in nursing facilities – a qualitative interview study among supervisor nurses

**DOI:** 10.2340/aos.v83.40686

**Published:** 2024-05-15

**Authors:** Hannaleena Jämsä, Marja-Liisa Laitala, Pirjo Kaakinen, Pekka Ylöstalo, Anna-Maija Syrjälä

**Affiliations:** a Research Unit of Population Health, University of Oulu, Oulu, Finland; b Research Unit of Population Health, Department of Cariology, Endodontology and Pedodontics, University of Oulu, Oulu, Finland; c Oulu University Hospital and Medical Research Center, Oulu, Finland; d Research Unit of Health Sciences and Technology, University of Oulu, Oulu, Finland; e Department of Oral and Maxillofacial Surgery, Oulu University Hospital, Oulu, Finland; f Research Unit of Population Health, Department of Periodontology and Geriatric Dentistry, University of Oulu, Oulu, Finland

**Keywords:** Supervisor nurse, qualitative study, older people, nursing home, antecedent

## Abstract

**Objectives:**

This qualitative study describes the views of supervisor nurses related to antecedents of oral care in Finnish nursing facilities.

**Methods:**

In the six largest cities in Finland, 19 supervisor nurses were interviewed and asked five semi-structured questions related to the antecedents of oral care in the units. The interviews were analyzed using inductive content analysis.

**Results:**

Qualitative content analysis revealed five main categories: awareness of nurses (3 categories, 9 subcategories), attitude and motivation (3 categories, 10 subcategories), supporting quality of life and health (4 categories, 11 subcategories), the meaning of oral health in the unit (3 categories, 10 subcategories), and the role of the supervisor nurse in oral health care (4 categories, 14 subcategories). The awareness of nurses regarding oral health was in most cases good. Nurses’ attitudes towards oral health and their motivation to oral care vary but were mostly good. Nurses were aware that oral care enhances the quality of life. The role of the supervisor nurse in organizing oral care was crucial.

**Conclusions:**

The performed analysis identified five main categories to describe antecedents for oral care in Finnish nursing facilities. The categories that needed to be improved were knowledge and attitude, and motivation related to oral care.

## Introduction

The older population is increasing and the number of people 80 years or older in the world is expected to triple from 2020 to 2050 [1]. The increasing number of dependent older people poses challenges for nursing staff in managing nursing care as well as maintaining their daily oral hygiene care. In Finland, approximately 38% of the 65–85-year-olds in 2021 were in nursing facilities for dependent older people [2].

According to a Finnish study, 44% of patients in long-term care institutions have been diagnosed with poor oral hygiene [3]. Furthermore, in institutional care, older people have been found to have more root caries [4, 5], as well as gingivitis and mild periodontitis [6]. Risk factors for oral diseases among older people include poor oral hygiene [6], reduced saliva production [4] and cariogenic diet [7].

Oral health among older people affects the quality of life [8, 9], nutrition [10], general health [11–13], physical frailty, functional disability, falls and hospitalization [14]. A crucial factor in maintaining oral health among the old residents in geriatric care units is good oral hygiene care managed by the nursing staff. Good oral hygiene care in older people prevents oral diseases [15], and respiratory infections, maintains oral function, and improves the quality of life [16] and psychosocial and functional wellbeing [15].

Oral hygiene care for an older person includes regular oral cavity inspection, removal of dental plaque, cleaning and moistening the mucous membranes and use of fluoride products [15]. The implementation of oral hygiene care in various geriatric care units has, however, been found to be insufficient [16, 17]. The poor implementation of oral care may be due to insufficient antecedents related to nurses as well as organization in the units. To implement oral care, nurses in care units should have a positive attitude towards oral care and possess the knowledge and skills to manage daily oral care practices for residents. Further, the care units should have adequate oral care equipment and the unit’s oral care instructions must be updated and aligned [15].

The antecedents to oral care in geriatric care units have been previously analyzed among care aides [18], nurses [19], and nurses and supervisors [20]. There is however, limited research that is focused specifically on the view of the supervisor nurses in an organization or the antecedents of oral care in the geriatric units. The view of the supervisor nurses is important to analyze because in care units, supervisor nurses are responsible for running everyday life in the units by managing finances, implementing practical nursing, and organizing the education and training of nursing staff [21].

In our previous qualitative study, we analyzed the role of supervisor nurses in the practical implementation of daily oral care for residents in private nursing homes and enhanced service housing units in Finland [22]. In that study, we found that oral care is reasonably well organized in these care units, and oral care of the residents is carried out daily with adequate dental equipment in most of the units [22]. In spite of fairly well functioning oral care, supervisor nurses highlighted the importance of oral health education and practical training in oral care in the units [22].

The aim of this qualitative study was to broaden the experiences and perspectives of the supervisor nurses in the provision of oral care in the units, and subsequently to clarify the antecedents of oral care as described by them. The study question was ‘What are the antecedents of oral care in private nursing homes and enhanced service housing units as described by supervisor nurses?’. The broader goals were to find possible development areas in the antecedents of oral care and to support supervisor nurses in developing the prerequisites and consequently improve the oral health of old residents in care units.

## Methodology

### Study design

This is a semi-structured qualitative interview study to analyze the antecedents of oral care as described by the supervisor nurses in private nursing homes and enhanced service housing units.

### Setting, sample and recruitment

Interviews were conducted in June–November 2021 on site in the care units. In every interview situation, there was only one interviewee and one interviewer. The total duration of the single interview was about 20 min. Nineteen supervisor nurses working in private nursing homes and enhanced service housing units in the six largest cities in Finland were recruited for the qualitative interview study. Participation in the interviews was voluntary. When recruiting, the first author (HJ) contacted the interviewees by phone and email, and there were no inclusion or exclusion criteria. In place, before the interviews, the interviewee and the interviewer signed consent for the study and the interviewees received written information about their rights and the progress of the research, as well as the use of the collected data.

A more detailed description of the study population has been published previously by Jämsä et al. 2023 [22].

### Data collection

The interview included five semi-structured themes about the antecedents of the units related to the oral health care of older people and the role of the supervisor nurse in arranging oral care. The themes were planned by a group of experienced professionals in nursing science, geriatric dentistry, and public health. The interviews were conducted by an experienced interviewer. In interviews, each question was asked once, and the interviewees were not prompted in any way by asking repeated questions, asking leading questions or asking to clarify the responses.

The interview was structured around the following themes:

1. The awareness of the staff of the unit about oral health care.
2. The significance of oral health in your unit.
3. The attitude and level of motivation related to taking care of oral health.
4. Supporting the quality of life and general health of older people with the help of oral care.
5. Your role in organizing oral health care in the unit.

Interviews were recorded and thereafter transcripted by the first author (HJ).

**Figure 1:**
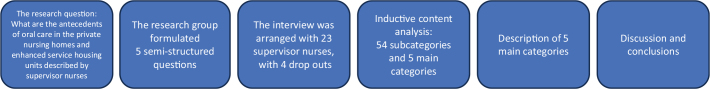
Study design.

The study design is shown in Figure 1.

### Ethical considerations

The Northern Ostrobothnia Hospital District gave approval for the voluntary and anonymous interviews on 7 January 2021 (298/2020).

### Data analysis

The analysis of the interviews followed the procedure of inductive content analysis [23]. The data from questions were transferred to a Word document matrix and interviews were transcribed verbatim into text format and checked for accuracy. No software was used in the analysis. First the author (HJ) got familiar with the transcripted data and made a comprehensive observation of the answers. The author read the answers several times, outlined them and studied, analyzed and reflected on them. The task of the author was to find and outline issues essential to the research themes. After becoming familiar with the answer, similar answers were reduced to codes. The reduced expressions were clustered based on the similarity of the content, abstracted into subcategories and main categories, and named based on content (Table 2). The coding and categorization of the data was done by three authors (HJ, PK, AS).

Each informant`s answer was coded with an ID number, and it is not possible to identify any informant. Personal information of all the participants was kept confidential, and their anonymity was ensured when reporting the results.

### Rigor of the analysis

The trustworthiness of the study is supported by the fact that each interview was conducted until saturation was achieved. Saturation of data refers to the point when sufficient answers were given by the study informants (19 supervision nurses), and there was no new information discovered anymore which means the researcher has collected enough data. Furthermore, the trustworthy of the study is supported by the fact that it is easy for the reader to follow the steps of the analysis and the formation of categories and by having direct quotes of the informants. The researchers experience with the subject under study strengthened the overall credibility of the study. However, when interpreting content analysis, one must take into account the possibility of social desirability, which potentially arises from being interviewed face-to-face and may skew the answers to be overly positive.

## Results

All supervisor nurses who participated in the interviews were women. Almost all were over 30 years old. More than two-thirds had more than 10 years of working experience as a nurse and more than half had worked in managerial positions for less than 10 years. Almost everyone brushed their own teeth twice a day and half of the nurses cleaned interdental spaces daily. Well over two-thirds visited the dentist regularly and had received personal guidance on oral care [22].

A total of five main categories described nurses’ experiences with oral care in private nursing homes and enhanced service housing units: awareness of nurses, attitude and motivation, supporting quality of life and health, meaning of oral health in the unit, and role of the supervisor nurse in oral health care (Table 1).

**Table 1:** Main categories and categories of content analysis.

Main category	Awareness of nurses	Attitude and motivation	Supporting quality of life and health	The meaning of oral health in the unit	Role of the supervisor nurse in oral health care
Category	The nurses have information about oral health care The level of knowledge varies between the units There is room for improvement in oral health care	Motivation and attitude towards oral health is good Motivation and attitude towards oral care varies Oral care is not done due to hurry and demands	Effect on general health Daily oral care Diet and mealtimes to support treatment Equipment and professional care	Part of the resident’s basic care Prevents general infections Affects the resident’s well-being	The role of the supervisor nurse in the implementation of oral care Coordinate visits by oral healthcare professionals Supports and encourages nurses Orientation and development of the skills Other duties

**Table 2:** Process of inductive content analysis.

Main category	Category	Subcategory
Awareness of nurses	The nurses have information about oral health care.	Importance of oral care. Nurses have expertise in oral care.
	The level of knowledge varies between the units	Staff has varying degrees of knowledge about oral care. Need for additional education for nurses on oral care. Some of the nurses are more aware of oral care. Nurses’ indifference towards oral care.
	There is room for improvement in oral health care.	Needs reminders about oral care. Oral care is gets overlooked amidst other tasks. There is room for improvement in the consideration of oral care.
Attitude and motivation	Motivation and attitude towards oral health are good.	Oral care is one of the basic responsibilities. Motivation is good for oral care. Nurses’ attitudes vary. Nurses thin oral care is important. Short documentation about oral care and counselling.
	Motivation and attitude towards oral care vary.	Additional education on the importance of oral care for overall health. Nurses are periodically reminded of the importance of oral care.
	Oral care is not done due to hurry and demands.	Daily dental care with residents can be challenging. Oral care is perceived as difficult. Oral care may not be done in a hurry.
Supporting quality of life and health	Effect on general health.	Preventing diseases and inflammations. Healthy mouth affects general health.
	Daily oral care.	Oral care is taken into account in daily activities. Promoting the residents’ quality of life. Teeth are taken care of daily.
	Diet and mealtimes to support treatment.	Oral care is taken into account after mealtimes. Eating affects the quality of life. Sugary snacks are not provided. Mealtimes have been carefully considered.
	Equipment and professional care.	Dental care equipment are checking regularly. Organizing visits by oral healthcare professionals.
The meaning of oral health in the unit	Part of the resident’s basic care	Part of the resident’s basic care. Affects the resident’s general health. Small part of the resident’s general health. Oral care must be taken into account.
	Prevents general infections	Oral care prevents additional diseases. Good oral care prevents infection. Oral care affects general health and reduces infections.
	Affects the resident’s well-being.	Impact on the resident’s quality of life and well-being. Oral care affects eating. Oral care is important for nutrition.
The role of the supervisor nurse in oral health care	Role of the supervisor nurse in the implementation of oral care	Responsible for the implementation of oral health care. Coordinates and directs. Doesn’t play a significant role in the organization of oral care. Role of supervisor nurse is small.
	Coordinate visits by oral healthcare professionals	Organizes visits to the dental hygienist. Organizes visits to the dentist. Organizes the dental check-ups. Organizes group visits.
	Supports and encourages nurses	Encourages to oral care. Supports the nurses in oral care. Participates in the residents’ dental care. Responsible for problematic situations.
	Orientation and development of skills	Responsible for the competence of the nurses. Responsible for the professionalism of the nurses.

### Awareness of nurses

As a rule, **nurses have an awareness** of oral health, and they have information about oral care, but the level of knowledge varies between the units. Most nurses have knowledge and know-how about the importance of oral care, but additional education is still needed especially in practical training on daily oral hygiene practices. Oral care for people with memory disorders is considered challenging, and the units would like to see more individual instruction related to oral care. Today, more attention is paid to oral care than before, and oral care in the units has been intensified; oral care is discussed in the units. However, some nurses are still indifferent to oral care and have room for improvement. Nurses need to be reminded of oral care from time to time because it is easy to forget it among other tasks.

*‘A clear change in the last 10 years. Clearly people have started to pay attention to the care and problems of the older people`s teeth’. [Participant 5]*

*‘It is certainly very clear to some but some downplay it’. [Participant 10]*

### Attitude and motivation

**Attitude and motivation** concerning oral health is mainly good. Oral care is one of the basic nursing care functions and nurses consider it important. Issues related to oral care are often left unrecorded by nurses and hence the working time used for it is not visible anywhere. This, too, highlights the importance of additional education and guidance. In some units, attitude and motivation towards oral care varies a lot. Oral care is neglected due to hurry and demands, and it is perceived as challenging.

*‘Maybe the attitude is good, but there would be room for improvement in making certain that it is taken into account’. [Participant 2]*

*‘Well, I think it is good, it is part of the overall treatment’. [Participant 4]*

*‘Oral care is easily the first thing left undone when one is in a hurry’. [Participant 7]*

### Supporting quality of life and health

The units’ aim was to consider the comprehensive well-being of the residents and consequently to **support residents’ quality of life and health**. Supervisor nurses and nursing staff know that good and regular oral care prevents systemic inflammation and diseases. Good oral care contributes to the quality of life of residents. Teeth are cleaned daily, and attention is given to oral care, especially after meals and after taking medication. Eating also affects the quality of life, and the mealtimes in the units have been carefully considered. Dental equipment is taken care of regularly and visits by dental professionals are organized regularly.

*‘As I understand it, if your teeth or dentures are well cared for on a daily basis, then of course it will improve the quality of life of any resident’. [Participant 8]*

*‘Well, it does affect how you are able to eat food, so it affects your quality of life; eating regular food is completely different from eating pureed food’. [Participant 9]*

### The meaning of oral health in the unit

As a rule, **the meaning of oral health care in the units** is considered to be important**.** Oral care is an important part of the basic care of the residents, and it prevents a general inflammatory condition and affects the well-being of residents. Almost all supervisor nurses mentioned that oral care affects general health, but one supervisor nurse felt that oral care is only a small part of a resident’s general health. Oral care is considered an important part of nutrition as it has a large impact on eating.

*‘Oral care is important for the treatment of other basic diseases’. [Participant 6]*

*‘It is one part of the rest of health. We try to prevent problems, to empower people as much as possible in daily activities’. [Participant 5]*

### The role of the supervisor nurse in oral health care

**The role of supervisor nurses was to be responsible** for the implementation of the unit’s oral care by coordinating and directing the operations. The supervisor books appointments with dentists and dental hygienists as well as possible group visits. An important role of the supervisor nurses is to support and encourage nursing staff to participate in the daily oral care of the residents, and provide oral care-related education and training. A few supervisor nurses themselves participate in caring for the residents’ teeth. The supervisor nurses are also responsible for any problematic situations and for orientating the unit’s nursing staff and ensuring their competence. They develop the unit’s operations and are responsible for the sufficient professionalism of the nursing staff. In addition, supervisor nurses are responsible for the unit’s resources and equipment procurement and, if necessary, work on oral care projects.

*‘To provide support for nursing tasks and ensure smooth operations, no matter what area it is’. [Participant 6]*

*‘I am responsible for all nursing work as well as organizing oral care’. [Participant 17]*

*‘My role is to encourage and remind the staff that to perform this oral care. Monitor as best as possible that it happens’. [Participant 10]*

In a few units, supervisor nurses reported that their role is not significant for oral care.

## Discussion

The performed analysis identified five main categories to describe antecedents for oral care, namely awareness of nurses, attitude and motivation, supporting quality of life and health, the meaning of oral health in the unit, and the role of the supervisor nurse in oral health care.

Although qualitative research does not aim to generalize, our results reflect previous results, as the nurses in our study, too, reported knowing that oral health is related to general health, but there is room for improvement in overall oral health-related knowledge [24] and attitudes to oral care [25]. One interesting finding in our study was that the supervisor nurses recognized their own responsibility in providing oral care, whereas in a previous study supervisor nurses felt that the role of nursing staff in oral care is limited [20].

The general trustworthiness of the study is supported by the fact that qualitative content analysis is a suitable research method for studying the antecedents for oral care described by supervisor nurses. The interview themes were unambiguous, comprehensible, and carefully planned by a group of experienced nursing and oral healthcare professionals which further support the trustworthiness of the study.

The strength of the study is that the respondents were of various ages and had various levels of working experience, which broadened the perspective of the study. The strengths of the study are also that the results of this study are morally defensible, and the disciplinary significance is important, because there is limited research about supervisor nurses’ perceptions concerning organizing oral care in geriatric units and they play an important role in organizing oral care. The results of the study can also be applied in practice, that is, the criterion of pragmatic obligation was met [26].

One limitation of this qualitative study is that it is built on semi-structured themes. An interview guide would have enabled a more open conversation, revealing additional perspectives in the interviews as compared to the semi-structured themes. Another limitation is that absolute truth in the interviews was probably not achieved during the study as most of the supervisor nurses did not participate in patient care in practice. Thus, supervisor nurses perhaps gave an overly positive picture of the implementation and antecedents of oral care in the units. Further, one weakness of the study is that the correlation between the background variables and responses of the participants could not be assessed due to the anonymity of the survey. The analysis of the correlation between the background variables and responses is warranted in future, in similar studies.

In Finland, The Nurses’ Study Guide may not specifically mention oral health care at all [27], and during nursing studies there seems to be limited education in oral health care. Thus, the role of supervisor nurse in organization of oral health related on-the-job training and education for nurses in care units is important in maintaining the oral health and oral health related quality of life of the residents.

The focus of the hypothesis generated from this qualitative study is the perception of responsibility of the supervisor nurses in the role of organizing oral care in the geriatric care units. This perception of responsibility is an essential starting point to having the teeth of residents cleaned daily and giving attention to oral care. A deeper analysis of the factors relating to the responsibility of supervisor nurses using a qualitative approach is warranted.

## Conclusion

The performed analysis identified five main categories to describe antecedents for oral care in private enhanced service housing units’ and nursing homes. Most categories seem to be fulfilled well; the categories that needed to be improved were knowledge and attitude and motivation related to oral care.

The results can be used to develop oral care among older people as well as to improve nurses’ oral health education in the nursing curriculum and on-the-job training. Nurses need support and encouragement from supervisor nurses to implement oral care in nursing homes and enhanced service housing units.

## Disclosure statement

The authors declare no conflict of interest.
